# Antimicrobial use and resistance data in human and animal sectors in the Lao PDR: evidence to inform policy

**DOI:** 10.1136/bmjgh-2021-007009

**Published:** 2021-12-01

**Authors:** Vilada Chansamouth, Mayfong Mayxay, David AB Dance, Tamalee Roberts, Rattanaxay Phetsouvanh, Bouakham Vannachone, Manivanh Vongsouvath, Viengmon Davong, Phout Inthavong, Syseng Khounsy, Bounxou Keohavong, Valy Keoluangkhot, Khamla Choumlivong, Nicholas PJ Day, Paul Turner, Elizabeth A Ashley, H. Rogier van Doorn, Paul N Newton

**Affiliations:** 1 Microbiology Laboratory, Mahosot Hospital, Vientiane, Lao People's Democratic Republic; 2 Lao-Oxford-Mahosot Hospital-Wellcome Trust Research Unit (LOMWRU), Mahosot Hospital, Vientiane, Lao People's Democratic Republic; 3 Centre for Tropical Medicine and Global Health, Nuffield Department of Medicine, University of Oxford, Oxford, UK; 4 Faculty of Postgraduate Studies, University of Health Sciences, Vientiane, Lao People's Democratic Republic; 5 Faculty of Infectious and Tropical Diseases, London School of Hygiene and Tropical Medicine, London, UK; 6 Department of Communicable Disease Control, Ministry of Health, Vientiane, Lao People's Democratic Republic; 7 Department of Livestock and Fisheries, Ministry of Agriculture, Vientiane, Lao People's Democratic Republic; 8 Department of Food and Drug, Ministry of Health, Vientiane, Lao People's Democratic Republic; 9 Infectious Disease Center, Mahosot Hospital, Vientiane, Lao People's Democratic Republic; 10 Setthathirath Hospital, Vientiane, Lao People's Democratic Republic; 11 Mahidol Oxford Tropical Medicine Research Unit (MORU), Faculty of Tropical Medicine, Mahidol University, Bangkok, Thailand; 12 Cambodia Oxford Medical Research Unit, Siem Reap, Cambodia; 13 Oxford University Clinical Research Unit, Hanoi, Viet Nam

**Keywords:** review, medical microbiology, epidemiology, public health

## Abstract

**Objectives:**

To review the scientific evidence base on antimicrobial use (AMU) and antimicrobial resistance (AMR) in human and animal sectors in the Lao PDR (Laos).

**Methods:**

We reviewed all publications from July 1994 (the first article describing AMR in Laos) to December 2020. Electronic searches were conducted using Google Scholar and PubMed with specific terms relating to AMR and AMU in Lao, French and English languages.

**Findings:**

We screened 1,357 peer-reviewed and grey reports by title and abstract and then full articles/reports. Of 80 included, 66 (83%) related to human health, nine (11%) to animal health, four (5%) to both animal and human health and one (1%) to the environment. Sixty-two (78%) were on AMR and 18 (22%) on AMU. Extended spectrum beta lactamase-producing *Escherichia coli* was the greatest concern identified; the proportion of isolates increased fivefold from 2004 to 2016 (2/28 (7%) to 27/78 (35%)) from blood cultures submitted to the Microbiology Laboratory, Mahosot Hospital, Vientiane. Carbapenem resistant *Escherichia coli* was first identified in 2015. Methicillin-resistant *Staphylococcus aureus* (MRSA) was uncommon, with 15 cases of MRSA from blood cultures between its first identification in 2017 and December 2020. AMR patterns of global antimicrobial resistance surveillance system (GLASS) target pathogens from livestock were less well documented. There were few data on AMU in human health and none on AMU in livestock. The first hospital AMU survey in Laos showed that 70% (1,386/1,981) of in-patients in five hospitals from 2017 to 2018 received antimicrobial(s). Antibiotic self-medication was common.

**Conclusion:**

AMR in Laos is occurring at relatively low proportions for some GLASS pathogens, giving the country a window of opportunity to act quickly to implement strategies to protect the population from a worsening situation. Urgent interventions to roll out new guidelines with enhanced one-health antibiotic stewardship, reduce antibiotic use without prescriptions, enhance surveillance and improve understanding of AMU and AMR are needed.

**Key questions**

**What is already known?**

- Irrational use of antimicrobials driving antimicrobial resistance (AMR) is a critically important issue worldwide.
- Data on antimicrobial susceptibility patterns of the global AMR surveillance system pathogens are commonly available in wealthier countries but fewer data are available in low-income and middle-income countries.
- There are scattered reports of AMR and antimicrobial use (AMU) in Laos, but they have not been synthesised to inform policy and implementation.

**What are the new findings?**

- Extended spectrum beta lactamase-producing *Escherichia coli* has become the greatest AMR concern in Laos.
- Carbapenem resistant *E. coli* and *Klebsiella pneumoniae* have been reported recently. Colistin resistant *E. coli and K. pneumoniae* have been reported in both livestock and a healthy individual.
- Data suggest a high proportion of hospital AMU in human health but no AMU data in animal health were found.

**What do the new findings imply?**

- The AMR situation in Laos is not as severe as in surrounding countries, suggesting that Lao has the opportunity to act quickly to control and combat AMR to avert a worsening situation.
- There is an urgent need for AMR and AMU monitoring in both hospitals and the community, improved antibiotic stewardship in humans and livestock and enhanced control of antibiotic use without prescriptions.
- More research is required to investigate appropriate solutions for the Lao context.

## Background

Antimicrobial resistance (AMR) is of major global public health concern, increasing in frequency for key pathogens and key antimicrobials. Evidence suggests that this is accelerated by antimicrobial use (AMU). AMR results in both direct consequences, such as longer duration of illness and hospital stay, and increased morbidity/mortality, and indirect consequences such as reduced productivity caused by sickness and increased family, community and health system economic burden.^1 2^ Since 2000, the number of antibiotics under development has fallen sharply.^3^ It has been estimated that approximately 700,000 people die every year from resistant pathogens, including bacterial infections, tuberculosis (TB), HIV and malaria.^4^ If AMR continues to increase with no appropriate action being taken, this could contribute 10 million excess deaths by 2050.^4^

Laos is a low- and middle-income country, bordered by Thailand, Cambodia, Vietnam, China and Myanmar. National action plans on AMR are available in all these countries, and Laos, and most of surrounding countries (except China and Vietnam) participate in the global antimicrobial resistance surveillance system (GLASS).^5^ However, in Laos, as is common elsewhere, different sectors (healthcare, agriculture, drug regulatory authorities) tend to work on their own strategies with little interaction. In October 2016, the Global Antibiotic Resistance Partnership-Laos committee was established as a Ministry of Health (MoH) technical working group to review the scientific evidence base to inform efforts to combat AMR in Laos, and provide evidence to the MoH AMR Surveillance and Control Committee. This report summarises the evidence base on AMU and bacterial resistance patterns in humans, animals and the environment in Laos, to identify important gaps and discusses the implications for health policy.

## Methods

### Information sources and inclusion/exclusion criteria

This narrative review focuses on the existing evidence on the prevalence of antimicrobial resistance among bacterial pathogens, particularly GLASS target pathogens, *Mycobacterium tuberculosis* and other available AMR pathogens in humans, animals and the environment in Laos. The review also describes data on AMU in these three sectors. All the peer-reviewed and grey literatures relating to Lao AMU and AMR data in humans, animals and the environment from 1994 (the year of the first published article on AMR in Laos) to 31 December 2020 in Lao, French and English languages were screened by title and abstract. We also included epidemiological studies, case studies and available raw data/reports from various governmental and non-governmental sectors in Laos.

This review does not include peer-reviewed or grey literatures relating to antimalarial, antiprotozoal, antihelmintic, antifungal and antiviral resistance.

### Published literature

Electronic searches were conducted using Google Scholar and PubMed. The search terms used were ‘Laos, Lao, antimicrobial susceptibility, antimicrobial resistance, antimicrobial use, animals, pharmacy, prescription, prevention, self-medication, environment, *Escherichia coli, Klebsiella pneumoniae, Acinetobacter baumannii, Staphylococcus aureus, Streptococcus pneumoniae, Salmonella* spp, *Shigella* spp, *Neisseria gonorrhoeae, Salmonella enterica* serovar Typhi, *Salmonella* Paratyphi, *Vibrio cholerae, Mycobacterium tuberculosis,* extended spectrum beta lactamase (ESBL) and methicillin-resistant *Staphylococcus aureus* (MRSA)’. Eligible articles were included after reading the abstracts. Full articles were then reviewed to obtain relevant data for inclusion.

### Grey literature

Relevant Lao Government departments were visited and data and reports requested. Annual reports, student project theses, results of projects on antibiotic use and resistance in Laos were requested from the Lao Food and Drug Department, Department of Healthcare and Rehabilitation, National Tuberculosis Centre, Microbiology Laboratory of Mahosot Hospital (the largest central hospital in Laos-the majority of laboratory data in this review were from this laboratory, which acts as a reference microbiology laboratory and provides services across the country), Livestock and Fisheries Department of Ministry of Agriculture & Forestry, Faculty of Agriculture of Lao National University and Institut de la Francophonie pour la Médecine Tropicale (IFMT).

We did not grade the quality of evidence, nor contact the authors for more information. The review results were described without further analysis.

### Patient and public involvement

There was no patient or public involvement in the planning or conduct of this work.

## Findings

**Figure 1:**
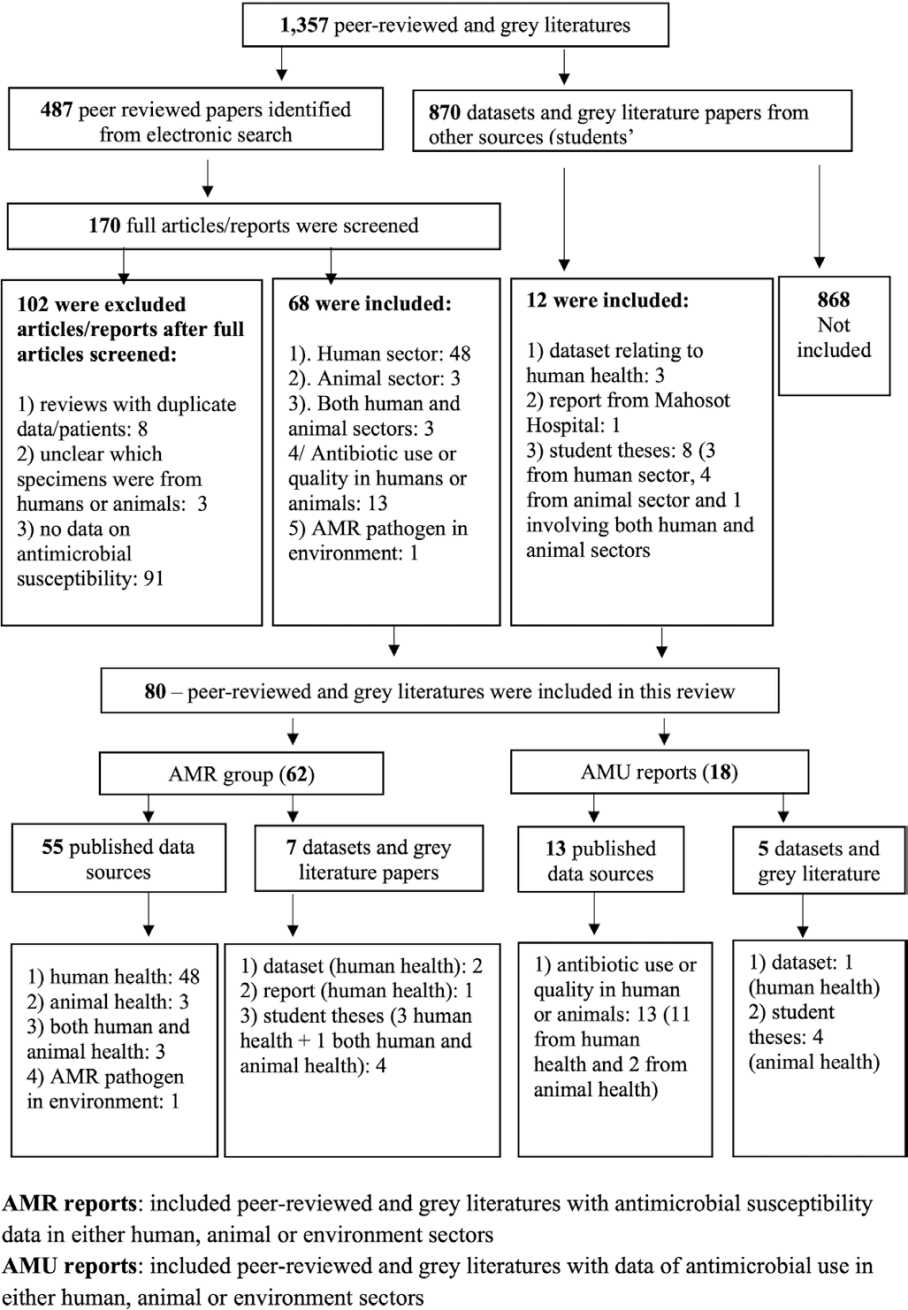
Search strategy and selection of studies for the review. AMR, antimicrobial resistance; AMU, antimicrobial use.

**Figure 2:**
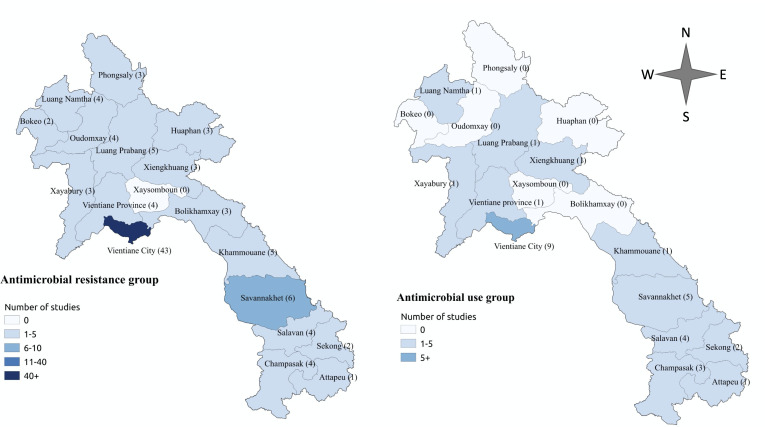
Distribution of antimicrobial resistance (AMR), left and antimicrobial use (AMU) data sources, right, in this review. Numbers after each province name (in parentheses) refer to the number of studies relating to either AMR or AMU in Laos. Numbers in parentheses were counted based on number of studies/reports relating to that province. Thirteen and two data sources were not included in the AMR and AMU maps, respectively, because their study sites were not clearly specified.

From 28 July 1994 to 31 December 2020, 1,357 peer-reviewed and grey literature reports were identified and screened by title and abstract; 487 (36%) were peer reviews from electronic searches and 870 (64%) were datasets or grey literature. After review, 80 peer-reviewed and grey literature reports were included. Of these, 62 (78%) were related to AMR and 18 (22%) to AMU in Laos (figures 1 and 2).

Of 80 data sources, 66 (83%) related to human health only, 9 (11%) related to animal health, 4 (5%) covered both human and animal health and one (1%) related to environmental AMR pathogens (online supplemental appendices 1–4). Forty-one (51%) were from, or related to, work done by the Lao-Oxford-Mahosot Hospital-Wellcome Trust Research Unit (LOMWRU), Mahosot Hospital, Vientiane.

10.1136/bmjgh-2021-007009.supp1 Supplementary data

Antimicrobial susceptibility testing (AST) described in this review was based on the clinical and laboratory standard institute (CLSI) methods, as was used in all microbiology laboratories in Laos until recently. It was replaced by the European Committee on AST system in mid-2019.^6^

### Evidence for AMR in bacteria infecting humans in Laos

From 1994 to 2020, *S. aureus, E. coli, K. pneumoniae* and *S*. Typhi were the most frequently mentioned bacterial pathogens in literature from Laos, mostly from fever aetiology studies with multiple pathogens isolated.

No distinction is made here between community-acquired infections (CAI) and hospital-acquired infections (HAI)^7^ as most available data sources did not include this information. The largest available dataset in this review was from Mahosot Hospital from 2000 to 2016, which did not include information to be able to identify HAI reliably. However, among 37,443 patients with blood cultures submitted to this laboratory during this period, blood cultures from 32,675 (87%) were submitted within 48 hours after admission to hospital; which are therefore believed to represent CAI. Blood cultures from 4,396 (12%) patients were submitted after 48 hours of admission. However, we cannot be sure that these represent HAI because of delays in submitting blood cultures, that only became an accessible service in 2000. In addition, 372 (1%) of patient records lacked dates of admission and/or blood cultures. It will be vital to systematically collect such data to increase our understanding of the comparative antimicrobial susceptibility patterns of both CAI and HAI.^8^

### AMR of WHO GLASS target pathogens

#### Escherichia coli

**Figure 3:**
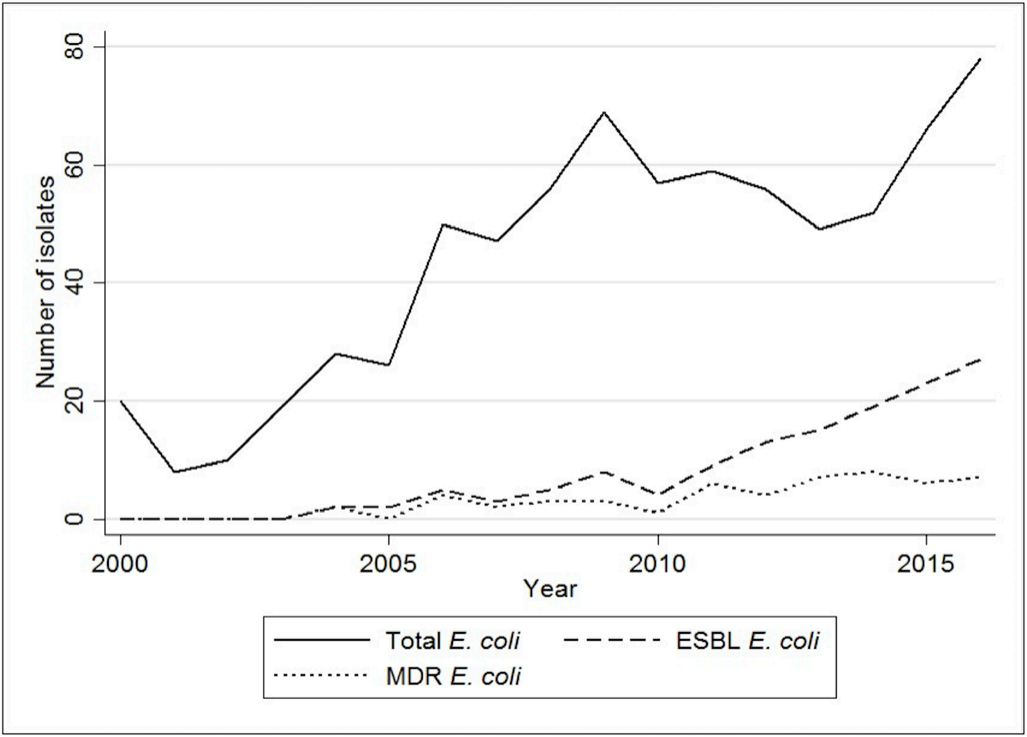
Trends of *Escherichia coli*, ESBL *E. coli* and MDR *E. coli* isolates from blood cultures from 2000 to 2016 at Mahosot Hospital, Vientiane. ESBL, extended spectrum beta lactamase; MDR, multidrug resistant.

ESBL-producing *E. coli* were first identified in Laos in 2004 from blood.^9^ Chang *et al.* reported the trend of ESBL-producing *E. coli* bacteraemia in Laos from 2010 to 2014, increasing from 7.8% (4/51) to 34.7% (17/49).^10^ A larger dataset from Microbiology/LOMWRU laboratory from 2004 to 2016, which included data from Chang *et al.*, showed that the actual proportion increased five-fold from 7% (2/28) in 2004 to 35% (27/78) in 2016 (figure 3).^8^ Among 280/1,837 inpatients and outpatients at Mahosot Hospital with urinary tract infection (UTI) that yielded significant growth from 2010 to 2011, 194/280 (69%) grew *E. coli*. Of these, 28/194 (14%) were confirmed as ESBL-producing. Only 22/151 (15%) were resistant to nitrofurantoin,^11^ which is not yet available in Laos. More recent (2017–2018) data on urine cultures from patients with UTI from Mahosot Hospital included 169 *E. coli* isolates, of which 90 (53%) were ESBL-producers, and 43 (25%) were multidrug resistant (MDR—non-susceptible to ≥1 agent in ≥3 antimicrobial categories).^12^ Nitrofurantoin resistance was found in 5% (8/169).^13^ *E. coli* carbapenem resistance was first identified in Laos in 2015 from a pus specimen from a Mahosot Hospital inpatient. By December 2020, nine more carbapenem resistant isolates from hospitalised patients had been identified by the Microbiology/LOMWRU laboratory (two from blood).^13–15^ New Delhi Metallo-betalactamase (NDM) genes were identified from four.^14^

#### Klebsiella pneumoniae

ESBL-producing *K. pneumoniae* were first identified in Laos from blood in 2000. Of 284 *K. pneumoniae* isolates from blood cultures at Mahosot Hospital from 2000 to 2016, 39 (14%) were ESBL producers and 20/39 (51%) were also MDR.^12^ Unlike ESBL-producing *E. coli*, ESBL-producing *K. pneumoniae* showed no clear time trend over this period and the proportion varied from 3% to 41% (online supplemental appendix 5).^8 10^ Of 32 *K*. *pneumoniae* isolates from blood during 2015 and 2016, 18 (56%) carried the CTX-M-15 gene.^16^ *K. pneumoniae* carbapenem susceptibility has been tested since 2010. Five resistant isolates were reported from non-blood specimens and one from blood between January 2018 and December 2020. Of these six, only three isolates were tested for carbapenemase and all of them carried NDM genes.^13^

10.1136/bmjgh-2021-007009.supp2 Supplementary data

#### ESBL-producing Enterobacterales carriage/colonisation and colistin resistance studies

ESBL-producing Enterobacterales stool colonisation has been investigated in Lao communities. ESBL-producing *E. coli* were found in 25% (100/397) of young children in 12 preschool childcare facilities in the capital city and the wider province of Vientiane.^17^ Of stool specimens from 57 healthy adults in southern Laos, 41 (72%) contained ESBL-producing *E. coli*.^18^ In a larger community cross-sectional study in a remote village in north-east Laos in 2016, ESBL-producing *E. coli* colonisation was found in 4% (11/268) of rectal specimens from healthy individuals.^19^ The most recent study published in 2021 demonstrated that 174/236 (74%) stool specimens collected from 20 healthy European visitors contained ESBL-producing Gram-negative bacteria during their stay in Laos. Of 292 ESBL-producing Gram-negative isolates from stool specimens, the CTX-M-15 gene was most frequently identified (262/292 (77%)).^20^

Although colistin is not available for human use in Laos, it is used for livestock.^21^ Eleven (6%) colistin resistant *K. pneumoniae* isolates were reported from 190 stool samples of healthy Lao individuals. Among these 11 isolates, nine different STs were reported and seven (64%) had *mgr*B genes without mutations.^22^ Of three isolates from Olaitan *et al.,*^22^ all three carried *mcr*-3 variants, and one carried both *mcr*-3 and *mcr*-8.^23^

#### Staphylococcus aureus

The first isolation of MRSA (*mecA* positive) in Laos was reported in 1997 from the nose of an inpatient, without signs of local infection, in a central Vientiane hospital.^24^ In 2002, the first two MRSA isolates from clinically infected patients were reported.^25^ The first MRSA isolated from blood was identified at Mahosot Hospital in 2017, with 14 subsequent cases by December 2020 for the whole country. *S. aureus* from non-blood culture specimens during 2012 and 2014 was described in 2017 with Panton-Valentine leukocidin positivity reported in 56/96 (58%) of isolates. MRSA was found in seven (7%) isolates including important strains circulating in neighbouring countries, such as ST239-MRSA-III and ST59/952-MRSA-V(T).^26^ More recent data during 2017 and 2018, yielded 95/619 (15%) MRSA from non-blood culture specimens submitted to the Microbiology/LOMWRU laboratory; 7/93 (8%) were resistant to trimethoprim-sulfamethoxazole, 10/90 (11%) to ciprofloxacin, 15/91 (16%) to chloramphenicol, 19/95 (20%) to gentamicin and 80/94 (85%) to tetracycline. No molecular investigations have been performed. All isolates were fully susceptible to vancomycin but this is not currently available in Laos.^13^

#### *Salmonella* Typhi

A case series of 913 *S*. Typhibacteraemic patients from Mahosot Hospital (2000–2018) included susceptibility data from diverse fever studies^27–32^; only 12/854 isolates (1.4%) were resistant to quinolones (either ciprofloxacin or nalidixic acid or both), 71/893 (8%) to ampicillin, 70/865 (8%) to chloramphenicol, 67/885 (7.6%) to trimethoprim–sulfamethoxazole and 59/848 (7%) were MDR. Ceftriaxone is the first choice of complicated typhoid treatment in Laos, and 91% (739/815) *S.* Typhi were highly susceptible to ceftriaxone. There has been no report of ceftriaxone resistant *S.* Typhi since 2009.^33^ Fluoroquinolone resistant *S*. Typhi in Laos are rare and treatment with fluoroquinolones are still likely to remain efficacious, as demonstrated in a clinical trial from 2002.^32^ Azithromycin is not routinely tested against *S.* Typhi in Laos. Azithromycin minimum inhibitory concentration (MIC) of ≤16 µg/mL was widely described as statistically clinical response for uncomplicated *S*. Typhi infection, 232/1,460 *S*. Typhi isolates from Laos were described in Parry *et al*. Of 1,460 isolates, 99.5% showed MIC of ≤16 µg/mL.^34^

#### Non-typhoidal *Salmonella*

Antimicrobial susceptibility of non-typhoidal *Salmonella* spp. (NTS) has not been documented in detail in Laos. Between 2000 and 2012, 168 NTS (63 from blood and 105 from faecal specimens) were isolated at Mahosot Hospital. NTS isolated from faecal samples were more frequently resistant to ampicillin and trimethoprim–sulfamethoxazole than those isolated from blood (p<0.05). In contrast, NTS resistant to ciprofloxacin were found more frequently in blood than in stool (p<0.001).^35^ In 2017, of 519 stool cultures reported from Mahosot Hospital, 35 grew NTS. Of these, 29% were resistant to azithromycin, 26% to trimethoprim-sulfamethoxazole, 23% to ciprofloxacin, 23% to ceftriaxone, 20% to chloramphenicol, 9% to ampicillin and 6% to nalidixic acid.^13^

#### *Shigella* spp.

*Shigella* spp. infection is also not well documented in Laos, with only two published reports.^36 37^ Forty-five *Shigella* isolates from Lao patient stools during 2006 and 2012, comprised 35 *S*. *flexneri*, nine *S*. *sonnei* and one *S. boydii*; and 14/45 (31%) were resistant to nalidixic acid and 34/45 (76%) were MDR. All isolates were susceptible to ceftriaxone and azithromycin.^37^

#### Neisseria gonorrhoeae

Four studies of *Neisseria gonorrhoeae* susceptibility in Laos have been published^38–41^ during this review period. In 2003, 1/131 (0.8%) *N*. *gonorrhoeae* isolates from 1937 genital clinical specimens was reported to be resistant to ceftriaxone, along with one further (0.8%) isolate with decreased spectinomycin susceptibility.^38^ Although no such strains have been described in subsequent studies, high frequencies of resistance to tetracycline, penicillin and ciprofloxacin were reported.^39–41^ More recent data from 2011 to 2015 included 158 *N*. *gonorrhoeae* isolated from 12 281 genital samples from patients at Mahosot Hospital; all were susceptible to ceftriaxone and spectinomycin. However, 99%, 90% and 85% were resistant to tetracycline, penicillin and ciprofloxacin, respectively.^41^ *N. gonorrhoeae* azithromycin susceptibility has not been tested for in Laos.

#### Streptococcus pneumoniae

Two publications have described *Streptococcus pneumoniae* infections in Laos,^42 43^ mainly focused on central nervous system infections, using overlapping datasets from Mahosot Hospital. Moore *et al.* described 2/23 isolates of *S. pneumoniae* from 2003 to 2009 from blood and cerebrospinal fluid (CSF), with reduced susceptibility to penicillin (both had minimum inhibitory concentrations≥0.12 µg/mL (0.39 and 0.125 µg/mL, respectively)).^42^ A larger dataset from 2003 to 2011 from the same hospital revealed that 3/11 isolates of *S. pneumoniae* from CSF had reduced susceptibility to penicillin.^43^

#### Acinetobacter baumannii

The first report on the susceptibility of *A. baumannii* in Laos was in 2019, describing 22 carbapenem-resistant *A. baumannii* isolates from 2017 (19 from endotracheal aspirates, two wound swabs and one blood culture). Of these, 18 (82%) were susceptible to amikacin but all were resistant to imipenem, ceftazidime, ciprofloxacin and tetracycline.^14 15^

#### Mycobacterium tuberculosis

The first multi-centre study of *Mycobacterium tuberculosis* susceptibility was conducted in 2010 in three Lao provinces. Of 87 (84%) *M*. *tuberculosis* cultured from 104 sputum samples, 8 (9.2%) were resistant to one or more antituberculous agents, of which seven were monoresistant to isoniazid (INH) and one was MDR that was subsequently found to be extensively drug resistant TB.^44^ Somphavong *et al.* characterised the genetics of these *M. tuberculosis* isolates from the national survey conducted from July 2010 to December 2011. Of 202 isolates, the East African-Indian lineage was the most common identified in all provinces, except Xiengkhuang. The Beijing lineage was found mostly in the northern and central Laos.^45^

From 2016 to 2017, of 1,006 sputum samples submitted to 42 TB laboratories throughout the country, 946 (94%) were GeneXpert MTB/RIF positive (897 (95%) new cases and 49 (5%) previously treated cases). Of these, 820 (87%) samples were available for AST. Seventy (8.5%) were resistant to antituberculous agents used for first-line treatment (either INH, rifampicin, ethambutol or streptomycin). Rifampicin resistance was found in 11/897 (1.2%) new cases and 2/49 (4%) previously treated cases. Five (0.6% of 820 isolates) were MDR (4/776 in new cases and 1/44 previously treated cases). No second-line TB drug (kanamycin, capreomycin and ofloxacin) resistance was reported.^46^

### AMR of other bacteria of local and regional importance

Diverse other infectious diseases are common in Laos,^47^ but there are very limited AMR data available. Intrinsic AMR is important for melioidosis and rickettsial diseases, reducing the spectrum of efficacious antibiotic therapy. *Burkholderia pseudomallei* is a common cause of bacteraemia in Laos and necessitates a prolonged treatment course of ceftazidime followed by co-trimoxazole, but acquired AMR has not become a clinical problem.^48^ Zoonoses such as rickettsial pathogens and leptospirosis are common causes of febrile diseases in Laos; the limited evidence also suggests that acquired AMR in these infections is not yet a major issue^48 49^ (online supplemental appendix 2).

### Evidence of AMR in bacteria in animals

Data on food production animal AMR in Laos are very limited, with the majority on *Salmonella* spp.,^50–54^ followed by *E. coli*,^19 53 55^ with sparser data for *Enterococcus faecalis* and *Enterococcus faecium*^56^ (online supplemental appendix 3). The proportion of *Salmonella* spp. contaminating pork/pig carcasses, beef and buffalos at slaughterhouses and retail markets in central and southern Laos ranged from 39% to 93% from 2007 to 2017, with the proportion of quinolone resistance at 2%–25%.^50–53^ Food production animals carrying ESBL-producing *E. coli* were also described in a remote rural village in Xiengkhuang Province in northern Laos. Of 252 food production animal rectal swabs, 21 (8%) carried ESBL-producing *E. coli*, even though these food production animals were mostly born in the village and fed with local products.^19^ Four colistin-resistant *E. coli* isolates from pigs have been described and also identified in a Lao boy with the same novel ST as his family’s pigs.^55^

### Evidence of AMU in Laos

AMU in Laos has also not been widely studied. Here we summarise ten AMU data sources for humans; one dataset and nine publications. Information on animal use is very limited, with only four veterinary student theses, one published report and one publication (online supplemental appendix 4).

#### AMU in humans

Antimicrobials were prescribed for between 45% and 70% of inpatients in Lao hospitals from 2004 to 2018.^57–60^ Doctors stated that they mainly prescribed antibiotics according to the National Standard Treatment Guidelines (STG), advice from their peers or from more experienced colleagues.^57^ Until recently these guidelines have not included detailed recommendations for antimicrobial prescribing for the diversity of Lao infectious diseases, but detailed MoH antimicrobial guidelines have now been released.^61 62^

From 2017 to 2018, five hospitals (Mahosot Hospital, Xiengkhuang, Luang Namtha, Vientiane and Salavan Provincial Hospitals) participated in the Global Point Prevalence Survey (http://www.global-pps.com) (Chansamouth *et al.* in prep). Of 1,981 hospitals charts screened, 1,386 (70%) patients received antimicrobial(s); the proportion of antimicrobial prescriptions was higher in provincial hospitals than in central hospitals at 72% *vs* 66% (p=0.003). Of 1,386 patients prescribed antimicrobial(s), 596 (43%) were prescribed two or more. Of all prescriptions, beta-lactams accounted for 1,272/2,052 (62%) (67% cephalosporins, 30% penicillins, 2% beta-lactam/beta-lactamase inhibitors and 1% carbapenems), metronidazole 287/2,052 (14%) and aminoglycosides 226/2,052 (11%) (online supplemental appendix 6 and 7).^59^ The appropriateness of the antimicrobials was not assessed. However, in Luang Namtha Provincial Hospital from 2008 to 2010, 560/1,095 (51%) patients received antibiotics but only 39/560 (7%) of these were considered appropriate.^27^ In an analysis of antibiotic use in 2019, 397/413 (94%) of patients were prescribed antibiotics for upper respiratory infection and 8/164 (4.9%) for common colds. First-line antibiotics based on Lao National STG (amoxicillin, ampicillin, erythromycin and penicillin V) were the most commonly prescribed.^60^

10.1136/bmjgh-2021-007009.supp3 Supplementary data

10.1136/bmjgh-2021-007009.supp4 Supplementary data

A large survey in Salavan Province between 2017 and 2018 revealed that among 796 participants from communities, 39% claimed that they used antibiotics during their previous illness and 22% admitted that they used antibiotics without indications or from informal sources.^63^ ‘Ampi’ or ampicillin was the most commonly mentioned antibiotic, by 76% of 775 survey participants, and wound treatment (44%) was the main reason for antibiotic use in Salavan in the same period.^64^ In Vientiane and Champasak Provinces, among 500 adults who self-medicated with antimicrobials for reproductive tract infections during the previous year, ampicillin (165 (33%)) was the most frequently used, and more than 50% used unrecommended combined medications.^65^ Participants claimed that seeing a doctor might not be necessary because they could easily access antibiotics without prescriptions and they obtain the same antibiotic that they had used previously.^66^ Pharmacies were common sources of antibiotics for self-medication (85% (326/384)) in three districts of Vientiane City in 2017.^67^ Moreover, some participants could access antibiotics from local grocery shops (69/384 (18%)).^67^ The use of antibiotics prior to hospital consultation, as indicated by antibiotic activity in urine, was significantly higher in children (60%) than in adults (47%) (p<0.0001).^68^ Ampicillin, amoxicillin, penicillin and tetracycline were frequently chosen for self-medication before deciding to go to hospital.^31 65 68^

More than one-third of Lao doctors from 25 public hospitals in four provinces thought that antibiotics in their hospitals were of poor quality.^57^ However, for consumers the cost of medications was of greater concern than their quality.^57 69^ Indeed, issues with substandard and falsified antibiotics have been described in Laos and these are likely to be globally neglected drivers of AMR and impaired patient outcome.^70 71^

#### AMU in animals

The use of antibiotics in Lao livestock is thought to be common but is not adequately supervised and there are few available data.^72 73^ Although no antimicrobial agents for animals have been registered with the Lao Department of Livestock and Fisheries (DLF), some antibiotics and vaccines are available at the DLF pharmacy and at private animal clinics. Most available publications/reports on AMU in animals focused on targeted treatment rather than growth promotion. Between 2002 and 2017, four students (out of 872) at the Department of Veterinary Medicine and DLF, National University of Laos studied the use of antibiotics in animals. These four theses focused on the effects of the use of cephalexin, amoxicillin, clavulanate, oxytetracycline, sulphonamides, enrofloxacin and gentamicin for treating infections in domestic dogs, with no studies on livestock.^74–77^ Among domestic elephants in Xayabury Province, antibiotics were used for treatment of abscesses, superficial wounds and eye problems. Oxyblue spray or penicillin–streptomycin were commonly used for abscesses and enrofloxacin for UTIs. As the country lacks provincial veterinary diagnostic laboratories, most infectious disease treatments in animals are empirical.^78^ A recent article on the antibiotic supply chain in the animal sector in Laos, but outside this review period,^21^ found that of 96 chicken farms and 96 pig farms, 49% and 60%, respectively, claimed that they gave antibiotics to their animals. Of 29 chicken farms with antibiotics found during the survey, tetracycline was the most commonly found, in 10/29 (34%) of chicken farms. Amoxicillin was the most common in pig farms at 17/73 (23%).^21^ There have not been reports or data on AMU in aquaculture, nor on AMR among fish bacterial pathogens in Laos.

#### AMR pathogens in the environment

There is almost no information on contamination of the Lao environment with AMR pathogens. In 2003, drug-resistant *Vibrio cholerae* from surface water samples was sought in thirteen tributaries of the Mekong River in cholera-epidemic areas in Laos after an outbreak in 2000. Twenty-two non-O1 and non-O139 *V. cholerae* isolates were tested against polymyxin B, tetracycline, ampicillin, ampicillin-clavulanic acid, erythromycin, nalidixic acid, chloramphenicol, trimethoprim–sulfamethoxazole and streptomycin. Polymyxin B resistance was found in 2/22 (9%) and ampicillin-resistance in 15/22 (68%), although isolates were susceptible to other tested antibiotics.^79^

## Discussion

There is little evidence on AMR awareness and how to engage with the public, health workers and policy makers in Laos.^57 58 80^ A large multicentre study of AMR awareness across 12 WHO member states (two from each WHO region) showed that the level of knowledge and awareness around appropriate antibiotic use varies from country to country.^81^ Many participants (32%–62%) thought that they should stop taking antibiotics when they felt better. Nearly half (43%) thought that they could buy the same antibiotics again if they had made them feel better during a previous illness and 44% believed that AMR is only a problem in people who take antibiotics regularly.^81^

Antimicrobial misuse in animal husbandry is thought to be one of the most important AMR drivers. However, we could not find any data on antibiotic use in livestock and aquaculture in Laos during this review period. There is an urgent need in Laos to systematically collect and analyse data on antibiotic use, consumption and resistance in humans, animals and the environment. Innovative public engagement strategies will be needed to raise awareness of optimal AMU and the risks of AMR.^82^

### AMR data compared with adjacent countries

Data on AMR among GLASS target pathogens are not well documented in Laos compared with neighbouring countries (tables 1 and 2). Most publications investigated causes of fever rather than specific pathogens and their susceptibility patterns. In Laos, current evidence suggests that the main concern is an increase in ESBL-producing *E. coli*. Community-acquired and hospital-acquired bacteraemia have not been well distinguished and further work to focus on this will be important. There is evidence for the emergence of carbapenem-resistant *E. coli* and *K. pneumoniae*, and colistin-resistant *K. pneumoniae* in Laos. Carbapenems and amikacin are the only antibiotic of choice for ESBL treatment available in Laos. It would be of great concern if combined carbapenem and colistin resistance were to emerge (colistin is not currently available in Laos for human use) as this would lead to infections that are essentially untreatable in Laos.

**Table 1:** Lao antimicrobial resistance frequency for *Escherichia coli,* isolated from blood and cerebrospinal fluid, compared with equivalent data from adjacent countries

*E. coli*	Laos*	Vietnam†^83^		Cambodia^85^	Thailand^84^
	Blood; n=750 (2000–2016)	All specimens; n=9,092 (2016–2017)	Blood and CSF; n=1,535 (2016–2017)	Blood; n=130 (2007–2010)	Blood; n=4,278 (2004–2010)
ESBL	135/693 (19%)‡	4,085/6,953 (59%)	655/1,107 (59%)	62 (48%)	
Amikacin	2/135 (1%)	4,188/8,785 (48%)	637/1,471 (43%)	5 (4%)	130/3,408 (4%)
Gentamicin	120/675 (18%)			73 (56%)	903/4228 (21%)
Cefotaxime	–	5,441/8,195 (66%)	931/1,402 (66%)	67 (52%)	885/3,892 (23%)
Ceftazidime	69/180 (38%)			47 (36%)	721/3,817 (19%)
Chloramphenicol	148/579 (26%)	–	–	–	18/72 (25%)
Ciprofloxacin	74/231 (32%)	5,813/8,682 (67%)	953/1,475 (65%)	85 (65%)	1,120/3,836 (29%)
Co-amoxiclav	94/673 (14%)	1,476/3,251 (45%)	180/577 (31%)	64 (49%)	1,139/3,910 (29%)
Imipenem	–	961/8,830 (11%)	116/1,483 (8%)	–	6/3,179 (<1%)
Meropenem	0/144 (0)			0	2/2,546 (<1%)
Trimethoprim–sulfamethoxazole	429/704 (61%)	5,704/7,843 (73%)	935/1,377 (68%)	214 (95%)	2,257/3,799 (59%)

Note that there are a paucity of published data and the year ranges differ between countries.

*LOMWRU data held by Microbiology/LOMWRU, Mahosot Hospital.

†Antimicrobial testing data from Vu *et al.*^83^ showed as a class of antibiotic rather than antibiotic agent.

‡Duration: 2004–2016; ESBL-producing *E. coli* in 2016 only was 27/78 (35%) in blood. However, ESBL-producing *E*. coli in urine (2017–2018) was 90/169 (53%).

CSF, cerebrospinal fluid; ESBL, extended spectrum beta lactamas; LOMWRU, Lao-Oxford-Mahosot Hospital-Wellcome Trust Research Unit.

**Table 2:** Lao antimicrobial resistance frequency for *Staphylococcus aureus,* isolated from blood and cerebrospinal fluid, compared with equivalent data from adjacent countries

*S. aureus*	Laos^89^	Vietnam*^83^		Cambodia^85^	Thailand^84^
	Blood; n=200 (2000–2011)	All specimens; n=4,833 (2016–2017)	Blood and CSF; n=715 (2016–2017)	Blood; n=46 (2007–2010)	Blood; n=1,881 (2004–2010)
MRSA	†	3,302/4,515 (73%)	476/674 (71%)	10/46 (22%)	357/389 (92%)
Ciprofloxacin	–	1,720/4,619 (37%)	297/689 (43%)	–	7/63 (11%)
Erythromycin	70/180 (39%)	3,861/4,661 (83%)	545/639 (79%)	24/46 (52%)	436/1,785 (24%)
Gentamicin	2/166 (1%)	1,674/4,090 (41%)	294/637 (46%)	–	106/719 (15%)
Penicillin	158/170 (93%)	2,347/2,400 (98%)	490/504 (97%)	45/46 (98%)	–
Tetracycline	39/81 (48%)	–		24/46 (52%)	–
Trimethoprim–sulfamethoxazole	16/151 (11%)	1021/4158 (25%)	233/661 (35%)	11/46 (24%)	341/1,828 (19%)
Vancomycin	0/52 (0)	45/2,680 (2%)‡	7/565 (1%)	–	6/1,380 (<1%)

Note that there are a paucity of published data and the year ranges differ between countries.

*Antimicrobial testing data from Vu *et al*^83^ showed as a class of antibiotic rather than antibiotic agent.

†The first MRSA bacteraemia in Laos was identified in 2017.

‡Resistant and intermediate.

CSF, cerebrospinal fluid; MRSA, methicillin-resistant *Staphylococcus aureus*.

MRSA is a universal public health concern, and well documented in neighbouring countries. In 2021, Vu *et al* reported that 3,302/4,515 (73%) of *S. aureus* in all specimen types from 13 hospitals in Vietnam during 2016 and 2017 were MRSA. In addition, of 674 *S*. *aureus* isolates from blood or CSF, 71% (476) were MRSA.^83^ In Thailand, the proportion of MRSA was high among patients with hospital-acquired *S. aureus* bacteraemia (48%, n=441), and 7% (n=1145) among community-acquired bacteraemia patients. The proportion of MRSA not susceptible to vancomycin (Vancomycin-resistant *Staphylococcus aureus* (VRSA)) was 0.4% (6/1,380).^84^ MRSA bacteraemia was also common in a large bacteraemia study (n=4,833 adult patients) in Phnom Penh, Cambodia; 22% (10/46) of *S. aureus* were MRSA but all were susceptible to vancomycin.^85^ In contrast, in Laos only six MRSA were isolated from blood during this review period; the number of MRSA in non-blood specimens was higher (15%). VRSA has not yet been reported in Laos.

These data suggest that Laos is thus surrounded by countries with an apparently higher frequency of antibiotic resistant pathogens. Paucity of AMR and AMU data puts Laos at a disadvantage in terms of understanding and controlling antibiotic resistance. However, the Lao data suggest that key bacterial pathogens within the country generally have a lower frequency of AMR than adjoining countries, especially *S. aureus* and *S*. Typhi, but that Laos has a severe and growing problem with ESBL production in *E. coli*, not only in patients but also in carriage by healthy individuals.^17–19^

Although there are no data to inform this objectively, Laos’ apparently lower burden of AMR may have resulted from historically lower antibiotic access and use due to being relatively isolated until ~20 years ago and financially impoverished, and there being only a small accessible commercial antibiotic market in the past. However, the apparent lower AMR burden may also partly reflect the paucity of systematic surveillance and research. Recent initiatives such as support for AMR and AMU surveillance by MoH through the Fleming Fund^86^ and KOICA^87^ aim to increase the availability of actionable data from across the country. Related initiatives such as the development of updated MoH infectious diseases treatment guidelines, AMR and AMU dashboards (https://www.youtube.com/watch?v=QELwHIPsKw4) will facilitate policy engagement and enhance antibiotic stewardship and AMR prevention, detection and pharmacist-led antimicrobial stewardship programmes and empowering hospital drugs and therapeutics committees could be explored as interventions to enhance AMU within healthcare facilities. Although it is illegal to sell antibiotics without a prescription in Laos, buying antibiotics without a prescription is anecdotally common and interventions to improve this situation will be vital. Recent interventions such as detailed antimicrobial treatment guidelines and inpatient and outpatient patient AMU data collection will provide an infrastructure for improving and monitoring AMU.

### Limitations

Data on AMU and AMR in Laos are sparse and scattered and are mostly from Vientiane City. The majority (49%) of data sources on AMR and AMU were from one site, at LOMWRU/ Mahosot Hospital. Though the number of publications on infectious diseases in Laos has greatly increased since 2000,^47 88^ there are few articles that specifically describe the AMU and AMR situations in Laos. Fifteen percent of the data sources on AMR and AMU in this review were not peer-reviewed. The majority of data were from epidemiological studies, cross-sectional studies and three large microbiology datasets from LOMWRU/Mahosot Hospital from 2000 to 2020.

## Conclusion

If AMR is, as these data suggest, of relatively low frequency in Laos for some GLASS target pathogens, it gives the country a key window of opportunity to act quickly to implement strategies to protect the population and health system from a worsening situation, through preventing, detecting and responding to AMR threats. However, the paucity of systematic surveillance of AMR and AMU in Laos must be taken into account when interpreting the low frequency of AMR in this setting. Enhanced coordinated and systematic AMU and AMR surveillance across the country is needed. Laos needs enhanced regulation of antibiotic availability, without compromising appropriate access, and strengthened antibiotic stewardship in health facilities and the community for systematic control and rationalisation of AMU, in order to prevent resistance before it spreads and becomes harder to manage. Strategies should also be put in place to control the use of colistin in livestock, as colistin is currently a last resort for treating key drug resistant pathogens. Specific strategies should also be developed to prevent a rise in *S. aureus* and *S*. Typhi resistance to key antibiotics and to reduce the high prevalence of ESBL-producing Enterobacterales.

## Data Availability

Data may be obtained from a third party and are not publicly available. All data relevant to the study are included in the article or uploaded asonline supplemental information.
