# Supplementary material for: Antimicrobial use and resistance data in human and animal sectors in the Lao PDR: evidence to inform policy
Source: BMJ Glob Health. 2021 Dec 1;6(12):e007009. doi: 10.1136/bmjgh-2021-007009 (PMC8638151; doi:10.1136/bmjgh-2021-007009)
Supplement: Supplementary data [file bmjgh-2021-007009supp001.pdf]

Supplementary appendix 1: Antimicrobial resistance data from 1994 to 2020 in Laos by WHO GLASS pathogens

**Study**	**Study period**	**Search**	**Study site**	**Pathogens**
Higa *et al*. 1994 (1)	1992 and 1993	Peer-reviewed	Vientiane City	*Staphylococcus aureus*
Higa *et al*. 1995 (2)	1992-1995	Peer-reviewed	Laos, no specific location	*Vibrio cholerae*, *Shigella* spp.
Mahosot Hospital 1995 (3)	1995	Report	Vientiane City	*Staphylococcus aureus, Enterococcus* spp., *Acinetobacter* spp., *Salmonella* Typhi, *Enterobacter cloacae, Gonococcus, Shigella flexneri, Shigella dysenteriae, Shigella sonnei,* EPEC, *Escherichia coli, Proteus mirabilis, Proteus vulgaris, Klebsiella oxytoca, Klebsiella pneumoniae,* Group D *Streptococcus, Pseudomonas aeruginosa, Pseudomonas* spp., *Candida* spp., *Staphylococcus saprophyticus*
Iwanaga *et al*. 1997 (4)	1995-1996	Peer-reviewed	Vientiane City	*Staphylococcus aureus*
Yamashiro *et al*. 1998 (5)	1996-1997	Peer-reviewed	Vientiane City	*Shigella flexneri, Shigella sonnei, Shigella boydii, Shigella dysenteriae, Shigella* spp., *Salmonella* spp., EPEC, ETEC, EIEC, EHEC, *Vibrio parahaemolyticus, Vibrio hollisae, Vibrio cholerae, Campylobacter jejuni* and *Campylobacter coli.*
Sithivong *et al*. 2002 (6)	1993-2001	Peer-reviewed	Vientiane City	*Staphylococcus aureus*
Kakinohaha *et al*. 2002 (7)	1998 and 2001	Peer-reviewed	Vientiane City	*Staphylococcus aureus*
Phantouamath *et al*. 2003 (8)	2000-2002	Peer-reviewed	Vientiane City	*Neisseria gonorrhoeae*
Hongsakhone 2004 (9)	2002-2004	Thesis	Oudomxay Province	*Salmonella* Typhi, *Escherichia coli* and Unidentified Gram-negative bacilli
Higa *et al*. 2004 (10)	2001-2002	Peer-reviewed	Vientiane City	*Staphylococcus aureus*
Phongmany *et al*. 2005 (11)	2001-2003	Peer-reviewed	Vientiane City	*Salmonella* Typhi

Phetsouvanh *et al*. 2006 (12)	2000-2004	Peer-reviewed	Vientiane City	Organisms isolated from blood culture: *Salmonella* Typhi, *Staphylococcus aureus, Escherichia coli, klebsiella pneumoniae, Burkholderia pseudomallei, Salmonella* spp., *Streptococcus pneumoniae,* and so on
Thammalangsy *et al*. 2006 (13)	2001-2002	Peer-reviewed	Vientiane City	*Neisseria gonorrhoeae, Chlamydia trachomatis, Candida* spp., *Bacterial vaginosis, Trichomonas vaginalis*
Sihavong *et al*. 2007 (14)	2000-2001	Peer-reviewed	Vientiane City	*Neisseria gonorrhoeae, Chlamydia trachomatis, Trichomonas vaginalis, Bacterial vaginosis, Candida* spp.
Bounsavath 2008 (15)	2003-2006	Thesis	Huaphan Province	*Salmonella* Typhi, *Escherichia coli, Klebsiella pneumoniae* and *Staphylococcus aureus*
Moore *et al*. 2010 (16)	2003-2009	Peer-reviewed	Vientiane City	*Streptococcus pneumoniae*
Elliot *et al.* 2012 (17)	2000-2011	Peer-reviewed	Vientiane City	*Staphylococcus aureus*
Stoesser *et al*. 2012 (18)	2004-2009	Peer-reviewed	Vientiane City	ESBL producing *Escherichia coli*
Phakhounthong *et al.* 2012 (19)	2010-2011	Thesis	Vientiane City	*Escherichia coli, Enterococcus faecalis, Klebsiella pneumoniae, Acinetobacter baumannii, Enterobacter cloacae, Staphylococcus aureus, Burkholderia pseudomallei, Pseudomonas aeruginosa, Proteus mirabilis, Gardnerella vaginalis, Streptococcus bovis* II, *Serratia marcescens, Streptococcus faecalis* B, *Staphylococcus* coagulase negative, *Salmonella* group D
Mayxay *et al.* 2013 (20)	2008-2010	Peer-reviewed	Luang Namtha and Salavan Provinces	*Salmonella* Typhi, *Escherichia coli, Burkholderia pseudomallei, Klebsiella pneumoniae, Staphylococcus aureus, Streptococcus suis, Streptococcus* Group A, *Streptococcus* Group C, *Salmonella enterica* Group C
Anderson *et al*. 2014 (21)	2000-2011	Peer-reviewed	Vientiane City	*Staphylococcus aureus, Escherichia coli, Klebsiella pneumoniae, Enterobacter aerogenes, Enterococcus faecalis, Streptococcus pyogenes, Streptococcus agalactiae, Streptococcus pneumoniae, Burkholderia pseudomallei, Acinetobacter baumannii, Listeria monocytogenes, Proteus mirabilis, Pseudomonas aeruginosa, Salmonella* spp.
Olaitan et al. 2014 (22)	2012-2013	Peer-reviewed	Laos, no specific location	*Klebsiella pneumoniae, Klebsiella oxytoca*
Parry *et al*. 2015 (23)	1995-2012	Peer-reviewed	Laos, no specific location	*Salmonella* Typhi, *Salmonella* Paratyphi

Stoesser *et al*. 2015 (24)	2011	Peer-reviewed	Vientiane City and Vientiane Province	Enterobacteriaceae
Nakayama *et al*. 2015 (25)	2012 and 2013	Peer-reviewed	Savannakhet Province	*Escherichia coli*
Mirabel *et al*. 2015 (26)	2006-2012	Peer-reviewed	Vientiane City	*Streptococcus pyogenes, Streptococcus oralis, Streptococcus mutans, Streptococcus anginosus, Streptococcus sanguinis, Streptococcus agalactiae, Escherichia coli, Enterococcus faecalis, Staphylococcus aureus,* coagulase negative staphylococci
Chansamouth *et al*. 2016 (27)	2006-2010	Peer-reviewed	Vientiane City	*Escherichia coli, Salmonella* Typhi, *Staphylococcus aureus, Klebsiella oxytoca*
Yeap *et al*. 2017 (28)	2012-2014	Peer-reviewed	Vientiane City	*Staphylococcus aureus*
Phuong *et al*. 2017 (29)	2000-2012	Peer-reviewed	Vientiane City	Non-typhoidal *Salmonella*
Darton *et al*. 2018 (30)	1994-2012	Peer-reviewed	Vientiane City	*Shigella flexneri* and *Shigella sonnei*
Pouangsouvanh *et al*. 2018 (31)	2011-2015	Peer-reviewed	Vientiane City	*Neisseria gonorrhoeae*
LOMWRU 2018 (32)	2018	Report	Laos, no specific location	NA
Rudge *et al.* 2019 (33)	2015-2016	Peer-reviewed	Vientiane City	*Streptococcus* spp., *Haemophilus influenzae, Klebsiella pneumoniae, Staphylococcus aureus, Escherichia coli*
Dubot-Peres *et al.* 2019 (34)	2003-2011	Peer-reviewed	Vientiane City	*Streptococcus pneumoniae, Mycobacterium tuberculosis, Escherichia coli, Streptococcus agalactiae, Neisseria meningitis,* Group C *Salmonella,* Group B *Salmonella,* Group D *Salmonella, Salmonella* Typhi, *Streptococcus suis, Klebsiella pneumoniae, Haemophilus influenzae* type B, *Burkholderia pseudomallei, Staphylococcus aureus, Morganella morganii*
Hadjadj *et al.* 2019 (35)	2012	Peer-reviewed	Laos, no specific location	*Klebsiella pneumoniae*
Roberts *et al*. 2020 (36)	2000-2018	Peer-reviewed	Laos, except Borkeo, Savannakhet, Champasak,	*Salmonella* Typhi

			Sekong and Attapeu	
Chang *et al*. 2020 (37)	2010-2014	Peer-reviewed	Laos, no specific location	*Escherichia coli*
Wyres *et al.* 2020 (38)	2015-2016	Peer-reviewed	Laos, no specific location	*Klebsiella pneumoniae*
Microbiology/LOMWRU (39)	2000-2016	Dataset	Vientiane City	Blood culture dataset – Multiple pathogens
Microbiology/LOMWRU (40)	2017-2019	Dataset	Vientiane City	Laboratory Information Management System (LIMS) – Multiple pathogens

Supplementary appendix 2: Antimicrobial resistance of additional bacteria of regional importance from 1994 to 2020 in Laos

Pathogens (references)	Details
Burkholderia pseudomallei	
Dance *et al.* 2014 (41)	*B. pseudomallei* resistant to trimethoprim-sulfamethoxazole were relatively rare in Laos with only 5/620 (0.8%) identified from all specimen types between 2003 and 2012 (41).
Microbiology/LOMWRU (40)	*B. pseudomallei* resistant to ceftazidime was firstly reported in 2017 from culture of a throat swab submitted to the Microbiology Laboratory, Mahosot Hospital (40).
Orientia tsutsugamushi	
Tantibhedhyangkul *et al.* 2010 (42)	Report of ciprofloxacin and ofloxacin resistance in vitro in *O. tsutsugamushi* isolates from Laos, probably representing innate resistance. This suggests that fluoroquinolones should not be used to treat scrub typhus (42).
Phuklia *et al.* 2019 (43)	Of 51 *O. tsutsugamushi* clinical isolates from Laos and Thailand, 45 isolates were from Laos and there was no evidence of *O. tsutsugamushi* resistance to doxycycline, azithromycin and chloramphenicol, but ofloxacin-resistance was detected (43).
***Leptospira* spp.**	
Boss *et al.* 2019 (44)	83 *Leptospira* spp. isolates from between 2006 and 2016, from patient samples submitted to the Microbiology Laboratory, Mahosot Hospital, were tested against azithromycin, ciprofloxacin, penicillin G, ceftriaxone, doxycycline and gentamicin by a novel disk diffusion method. The median zone sizes were 85, 70, 70, 69, 56 and 35 mm, respectively. The results suggested that drug resistance to these six antimicrobials has not yet occurred in Lao *Leptospira* spp. (44).
Vibrio cholerae	
Higa *et al*. 1995 (2)	Antimicrobial susceptibility of *V. cholerae* was first described in 1995 in Laos with strains fully susceptible to all tested antimicrobials (ampicillin, tetracycline, erythromycin, ofloxacin and polymyxin) (2).
Iwanaga *et al.* 2000 (45)	Of 99 *Vibrio cholerae* isolates from an outbreak in Laos in 1998; 95 (96%) were resistant to tetracycline (45).
Phantouamath *et al.* 2001 (46)	209 *V. cholerae* O1 isolates from 1993-1999 were tested for susceptibility to ampicillin, erythromycin, ofloxacin, nalidixic acid, chloramphenicol, tetracycline, sulfamethoxazole-trimethoprim and polymyxin B. Over these 7 years, the overall susceptibility patterns for ampicillin, erythromycin, ofloxacin and nalidixic acid changed little. Isolates were highly resistant to sulfamethoxazole-trimethoprim and moderately resistant to tetracycline in 1998 (46).
Iwanaga *et al*. 2004 (47)	

	Antibiotic resistant genes of 50 *V. cholerae* O1 isolates from 1993-2000 were described. Strains before 1997 carried a class I integron with an *aadA1* gene cassette but an SXT constin was found after 1997 as the reemerged El Tor O1 strains (47).
***Streptococcus* pyogenes**<br>(Group A *Streptococcus*)<br>Rattanavong *et al*. 2016 (48)	Of 124 *Streptococcus pyogenes* isolates cultured between 2004 and 2013 from the Microbiology Laboratory, Mahosot Hospital, all isolates were fully susceptible to penicillin. Only 7% of S. pyogenes was resistant to chloramphenicol, 8% resistant to erythromycin and <1% resistant to ofloxacin (48).
Helicobacter pylori<br>Vannarath *et al.* 2016 (49)	During 2010 and 2012, 119 dyspeptic patients with *H. pylori* infection were tested for clarithromycin and fluoroquinolone resistance using a GenoType®HelicoDR test. Of these, 15 (12.6%) were resistant to clarithromycin and 16 (13%) resistant to fluoroquinolones (49).
Clostridium difficile<br>Cheong *et al*. 2017 (50)	Of 70 stool specimens submitted to the Microbiology Laboratory, Mahosot Hospital between September and October 2013, five (7%) samples grew *C. difficile*. All five were susceptible to moxifloxacin, metronidazole and vancomycin, whereas four (80%) were intermediately susceptible to clindamycin and one (20%) was resistant to clindamycin (50).
Neisseria meningitidis<br>Batty *et al.* 2020 (51)	Over 11 years in three laboratories in Southeast Asia (Thailand, Laos and Cambodia), 8/23 (44%) of *N. meningitidis* isolated from blood or CSF were resistant to chloramphenicol, 11/23 (48%) had reduced susceptibility to penicillin, 1/23 (4%), whilst all were susceptible to ceftriaxone (51).
Mycobacterium tuberculosis<br>Iem *et al.* 2013 (52)<br>Iem *et al.* 2019 (53)<br>Somphavong *et al.* 2019 (54)	More details in the main text

Supplementary appendix 3: Evidence of antimicrobial resistance from 1994 to 2020 in animals in Laos

Pathogens (references)	Samples	Sites	Antimicrobial susceptibility
***Salmonella* spp.**			
Boonmar *et al.* 2008 (55)	47 pigs	slaughterhouse in Vientiane	37/47 (76%), carried *Salmonella* spp. Two percent were resistant to nalidixic acid and 12% were MDR (defined as resistant to three or more of ampicillin, tetracycline, chloramphenicol, streptomycin and trimethoprim-sulfamethoxazole) (55).
Boonmar *et al.* 2013 (56)	17 beef, 27 pork, 5 buffalo meat	retail markets in Pakse District, Champasak, Province	4/17 (82%), 25/27 (93%) and 4/5 (80%) of beef, pork and buffalo meat samples, respectively carried *Salmonella* spp.. All available isolates (60/80-75%) were susceptible to ciprofloxacin and norfloxacin, cefotaxime (94-100%) and nalidixic acid (86-100%) but many were resistant to ampicillin (60-70%), tetracycline (60-75%) and streptomycin (57-80%) (56).
Sinwat *et al.* 2016* (57)	112 pork, 137 pig carcasses and 129 rectal swabs of pig carcasses	fresh markets, Savannakhet Province and Vientiane City	72% (81/112) of pork meat samples, 53% (73/137) from pig carcasses and 39% (50/129) from rectal swabs of pig carcasses from fresh markets, carried *Salmonella* spp.. Of 237 *Salmonella* spp. isolates from 378 specimens, 10% (24/237) were resistant to nalidixic acid (57).
Sunn (MSc thesis) (58)	140 pig rectal swabs and pork carcasses	Vientiane City	86/140 (61%) carried *Salmonella* spp., 1 (1%) was resistant to colistin, 2 (2%) to ceftazidime, 2 (2%) to cefotaxime and 2 (2%) to cefpodoxime (the article did not state whether these two resistant isolates were the same isolates and both were resistant to all three cephalosporins). Of these two cefpodoxime resistant isolates, one isolate was confirmed as ESBL producing (58).
Escherichia coli			
Chang 2016* (59)	252 swabs from healthy domestic animals (chicken, dogs, cats, cows, goats, pigs and horse)	Xiengkhuang Province	21/252 (8%) grew ESBL-producing *E. coli*. Of these 21, one animal sample grew *E. coli* and *K. pneumoniae* that were both ESBL producing. Among 21 ESBL-producing *E. coli*, all were fully susceptible to amoxicillin-clavulanic acid, meropenem and amikacin, with a spectrum of resistance to ceftazidime (29%), gentamicin (48%), tetracycline (67%), ciprofloxacin (43%), chloramphenicol (14%) and trimethoprim-sulfamethoxazole (43%). These domestic animals were mostly born in the village and were fed with local products (59).
Sunn (MSc thesis) (58)	140 swabs of pig and pork carcasses	Vientiane City	133/140 (95%) grew *E. coli* and resistance to colistin was found in 38 (28%), to ceftazidime in 4 (3%), to cefotaxime in 9 (7%) and ESBL producing *E. coli* were found in 6 (4%) (58).
Olaitan *et al.* 2015* (60)	Stool specimens of 190 healthy humans, 44 goats and 18 pigs	rural Laos	four colistin-resistant *E. coli* from pigs; all of them belonged to novel STs. All ten colistin-resistant *E. coli* isolates contained the *mcr-1* gene. A boy carried colistin-resistant *E. coli* with

			the same novel ST as his family's pig and the boy had had no exposure to colistin but fed the pig with no personal protection (60).
Enterococcus faecalis* and *Enterococcus faecium<br>Thu *et al.* 2019* (61)	80 rectal swabs of pigs, 80 swabs of pig carcasses and 72 meat swabs	Savannakhet Province and Vientiane City	Of 143 *E. faecium* isolates from pigs/pig carcasses/pork, 13 (9%) were resistant to ampicillin, 12 (9%) to chloramphenicol, 2 (1%) to gentamicin, 79 (55%) to tetracycline. Whilst, 30 *E. faecalis* isolates from pigs/pig carcasses/pork were fully susceptible to ampicillin, but 13 (43%) were resistant to chloramphenicol, 19 (63%) to gentamicin and 28 (93%) to tetracycline (61).

* described antimicrobial resistance data in both human and animal

Supplementary appendix 4: Data sources of antimicrobial use in human and animal from 1994 to 2020 in Laos.

Study	Study period	Sector	Study site	Antimicrobials and details investigated
Syhakhang *et al*. 2004 (62)	1997 and 1999	Human health	Savannakhet Province	Ampicillin, tetracycline, chloroquine
Keohavong *et al*. 2006 (63)	2003-2004	Human health	Laos, no specific location	Rational use of antimicrobials
Sihavong *et al*. 2006 (64)	2002	Human health	Vientiane City and Champasak Province	Understanding and use of health information among adult population self-medicating with reproductive tract infection
Khennavong *et al.* 2011 (65)	2003-2004 & 2005	Human health	Vientiane City and Savannakhet Province	Urine investigation for previous antimicrobial usage
Sihavong *et al.* 2011 (66)	2002-2006	Human health	Vientiane City and Champasak Province	Treatment seeking behaviour for sexually transmitted infections - qualitative study
Quet *et al.* 2015 (67)	2012	Human health	Khammuane, Luang Prabang, Sekong Provinces, Vientiane City	Knowledge, attitude and practice of antibiotic prescriptions of Laos doctors
Phonlavong and Kitikannakorn 2018 (68)	2017	Human health	Vientiane City	Knowledge, beliefs and care seeking behaviour about antibiotics
keohavong *et al.* 2019 (69)	2016	Human health	Savannakhet Province	Assess patterns of antibiotic prescriptions among children under 5
Tabernero *et al.* 2019 (70)	2012	Human health	Savannakhet, Salavan, Sekong, Champasak and Attapeu Provinces	Assess the availability and quality of antimalarials and antibiotics in private sectors in five southern provinces of Laos
Haenssgen *et al.* 2019 (71)	2017-2018	Human health	Salavan Province	Antibiotic-related knowledge, attitude and practice in Salavan Province, Laos
Haenssgen *et al.* 2020 (72)	2017-2018	Human health	Salavan Province	Assess the relationship between precarity, other forms of deprivation and healthcare-seeking behaviour in Salavan Province, Laos

LOMWRU database (73)	2017-2018	Human health	Vientiane City, Salavan, Vientiane, Xiengkhuang, Luang Namtha Provinces	Proportion of hospital antimicrobial prescription and most common prescribed antimicrobials
Labatut and uter 2010 (74)	2007-2009	Animal health	Sayabury Province	Use of antibiotics in elephants in Laos (oxytetracycline, penicillin-streptomycin, Negasunt (including sulfonamide), mebendazole, Dufamec (ivermectin + praziquantel), and Archifen (chloramphenicol))
Theungphachan 2012 (75)	2012	Animal health	Laos, no specific location	Report (including list of available antimicrobials in animal sector)
Phanthavong 2016 (76)	2016	Animal health	Vientiane City	Use of antibiotics in pets (cephalexin, amoxicillin and clavulanate)
Innoula 2017 (77)	2017	Animal health	Vientiane City	Use of antibiotics in pets (enrofloxacin and gentamicin)
Boudakham 2017 (78)	2017	Animal health	Vientiane City	Use of antibiotics in pets (amoxicillin and Oxy-Mycin (oxytetracycline))
Keovilay 2017 (79)	2017	Animal health	Savannakhet Province	Use of antibiotics in pets (enrofloxacin and sulfonamides)

References

1. Higa N, Sithivong N, Iwanagal M. A comparative study on *Staphylococcus aureus* isolated in Lao PDR and in Japan. *Jpn J Trop Med Hyg*. 1994;22(3):129-31.
2. Higa N, Iwanaga M, Utsunomiya A, et al. Drug sensitivity of *Vibrio cholerae* and *Shigella* species in the world. *Jpn J Trop Med Hyg*. 1995;23(3):159-64.
3. Microbiology/Mahosot. Résultats des cultures et antibiogrammes. Microbiology report. Microbiology Laboratory, Mahosot Hospital 1995.
4. Iwanaga M, Sisavath L, Higa N, et al. Emergence of methicillin resistant *Staphylococcus aureus* in Laos. *Jpn J Trop Med Hyg*. 1997;25(3):103-6.
5. Yamashiro T, Nakasone N, Higa N, et al. Etiological study of diarrheal patients in Vientiane, Lao People's Democratic Republic. *J Clin Microbiol*. 1998;36(8):2195-9.
6. Sithivong N, Phantouamath B, Chomlasak K, et al. Monitoring of drug resistant *Staphylococcus aureu*s in People's Democratic Republic of Lao. *Jpn J Trop Med Hyg*. 2002;30(2):109-13.
7. Kakinohana S, Uemura E, Insisiengmay S, et al. Staphylococcus aureus isolated from hospital staff: a comparative study of Laos and Japan. *J Infect Chemother*. 2002;8(4):336-40.
8. Phantouamath B, Sithivong N, Phouthavanh T, et al. Drug susceptibility of *Neisseria gonorrhoeae* in Lao People's Democratic Republic. *Jpn J Trop Med Hyg*. 2003;31(3):117-20.
9. Hongsakhone S. Fievre typhoid, etude epidemio – Clinique et profil de sensibilite a l'hopital provincial d'Oudomxay, Laos – 4e promotion, 2002-2004 [MSc]. IFMT: IFMT; 2004.
10. Higa N, Sithivong N, Phantouamath B, et al. Initial stage of hospital contamination with methicillin-resistant *Staphylococcus aureus* in Lao People's Democratic Republic. *J Hosp Infect*. 2004;56(2):125-30.
11. Phongmany S, Phetsouvanh R, Sisouphone S, et al. A randomized comparison of oral chloramphenicol versus ofloxacin in the treatment of uncomplicated typhoid fever in Laos. *Trans R Soc Trop Med Hyg*. 2005;99(6):451-8.
12. Phetsouvanh R, Phongmany S, Soukaloun D, et al. Causes of community-acquired bacteremia and patterns of antimicrobial resistance in Vientiane, Laos. *Am J Trop Med Hyg*. 2006;75(5):978-85.
13. Thammalangsy S, Sihavong A, Phouthavane T, et al. The prevalence of lower genital tract infections among ante-natal care (ANC) clinic patients in two central hospitals, Vientiane, Lao People's Democratic Republic. *Southeast Asian J Trop Med Public Health*. 2006;37(1):190-9.
14. Sihavong A, Phouthavane T, Lundborg CS, et al. Reproductive tract infections among women attending a gynecology outpatient department in Vientiane, Lao PDR. *Sex Trans Dis*. 2007;34(10):791-5.

15. Bounsavath N. Etiology of septicemia in Houaphan Province Hospital 2005-2008 [Internal medicine]. University of Health Sciences: University of Health Sciences, Laos; 2008.
16. Moore CE, Sengduangphachanh A, Thaojaikong T, et al. Enhanced determination of *Streptococcus pneumoniae* serotypes associated with invasive disease in Laos by using a real-time polymerase chain reaction serotyping assay with cerebrospinal fluid. *Am J Trop Med Hyg*. 2010;83(3):451-7.
17. Elliott I, Phommasone K, Vongsouvath M, et al. *Staphylococcus aureu*s Bacteraemia in the Lao People's Democratic Republic: Antibiotic Susceptibility Patterns and Clinical Management. *Lao Medical Journal*. 2012;3(1):3-15.
18. Stoesser N, Crook DW, Moore CE, et al. Characteristics of CTX-M ESBL-producing *Escherichia coli* isolates from the Lao People's Democratic Republic, 2004-09. *J Antimicrob Chemother*. 2012;67(1):240-2.
19. Phakhounthong K, Sivanthong P, Sisomboun P. Etiological pathogens and their antibiotic susceptibility pattern among adult patients with urinary tract infections at Mahosot Hospital [Medical doctor]. Available at University Library: University of Health Sciences; 2012.
20. Mayxay M, Castonguay-Vanier J, Chansamouth V, et al. Causes of non-malarial fever in Laos: a prospective study. *Lancet Glob Health*. 2013;1(1):e46-54.
21. Anderson M, Luangxay K, Sisouk K, et al. Epidemiology of bacteremia in young hospitalized infants in Vientiane, Laos, 2000-2011. *J Trop Pediatr*. 2014;60(1):10-6.
22. Olaitan AO, Diene SM, Kempf M, et al. Worldwide emergence of colistin resistance in *Klebsiella pneumoniae* from healthy humans and patients in Lao PDR, Thailand, Israel, Nigeria and France owing to inactivation of the PhoP/PhoQ regulator mgrB: an epidemiological and molecular study. *Int J Antimicrob Agents*. 2014;44(6):500-7.
23. Parry CM, Thieu NT, Dolecek C, et al. Clinically and microbiologically derived azithromycin susceptibility breakpoints for *Salmonella* enterica serovars Typhi and Paratyphi A. *Antimicrob Agents Chemother*. 2015;59(5):2756-64.
24. Stoesser N, Xayaheuang S, Vongsouvath M, et al. Colonization with *Enterobacteriaceae* producing ESBLs in children attending pre-school childcare facilities in the Lao People's Democratic Republic. *J Antimicrob Chemother*. 2015;70(6):1893-7.
25. Nakayama T, Ueda S, Huong BT, et al. Wide dissemination of extended-spectrum beta-lactamase-producing *Escherichia coli* in community residents in the Indochinese peninsula. *Infect Drug Resist*. 2015;8:1-5.
26. Mirabel M, Rattanavong S, Frichitthavong K, et al. Infective endocarditis in the Lao PDR: clinical characteristics and outcomes in a developing country. *Int J Cardiol*. 2015;180:270-3.
27. Chansamouth V, Thammasack S, Phetsouvanh R, et al. The Aetiologies and Impact of Fever in Pregnant Inpatients in Vientiane, Laos. *PLoS Negl Trop Dis*. 2016;10(4):e0004577.
28. Yeap AD, Woods K, Dance DAB, et al. Molecular Epidemiology of *Staphylococcus aureus* Skin and Soft Tissue Infections in the Lao People's Democratic Republic. *Am J Trop Med Hyg*. 2017;97(2):423-8.
29. Phuong T, Rattanavong S, Vongsouvath M, et al. Non-typhoidal Salmonella serovars associated with invasive and non-invasive disease in the Lao People’s Democratic Republic. *Trans R Soc Top Med Hyg*. 2017;111(9):418-24.

30. Darton TC, Thanh Tuyen H, Chung The H, Newton PN, Dance DAB, Phetsouvanh R, et al. Azithromycin resistance in *Shigella* spp. in Southeast Asia. Antimicrob Agents Chemother. 2018.
31. Phouangsouvanh S, Mayxay M, Keoluangkhot V, et al. Antimicrobial susceptibility of *Neisseria gonorrhoeae* isolates in Vientiane, Lao PDR. *J Glob Antimicrob Resist*. 2018;13:91-3.
32. LOMWRU. Scientific Annual Report for 2018. 2018 [Access date 09 April 2021]. Available from: https://www.tropmedres.ac/_asset/file/lomwry-2018-annual-report-to-lao-moh.pdf
33. Rudge JW, Inthalaphone N, Pavlicek R, et al. Crrection: "Epidemiology and aetiology of influenza-like illness among households in metropolitan Vientiane, Lao PDR": A prospective, community-based cohort study. *PloS One*. 2019;14(4):e0216491.
34. Dubot-Pérès A, Mayxay M, Phetsouvanh R, et al. Management of Central Nervous System Infections, Vientiane, Laos, 2003–2011. *Emerg Infect Dis*. 2019;25(5):898.
35. Hadjadj L BS, Olaitan AO, et al. Co-occurrence of Variants of mcr-3 and mcr-8 Genes in a Klebsiella pneumoniae Isolate From Laos. *Front Microbiol*. 2019;10:2720.
36. Roberts T, Rattanavong S, Phommasone K, et al. Typhoid in Laos: An 18-Year Perspective. *Am J Trop Med Hyg*. 2020;102(4):749.
37. Chang K, Rattanavong S, Mayxay M, et al. Bacteremia Caused by Extended-Spectrum Beta-Lactamase-Producing Enterobacteriaceae in Vientiane, Lao PDR: A 5-Year Study. *Am J Trop Med Hyg*. 2020;102(5):1137-43.
38. Wyres KL NT, Lam MMC, et al. Genomic surveillance for hypervirulence and multi-drug resistance in invasive Klebsiella pneumoniae from South and Southeast Asia. *Genome Med*. 2020;12(1):11.
39. Microbiology/LOMWRU. Data from: Undetermined infections (UI) 2000-2016. Microbiology Laboratory/Lao-Oxford Mahosot Hospital Wellcome Trust Research Unit (LOMWRU), Mahosot Hospital, Vientiane, Laos; 2016.
40. Microbiology/LOMWRU. Data from: Laboratory Information Management System (LIMS) 2017-2020. Microbiology Laboratory/Lao-Oxford Mahosot Hospital Wellcome Trust Research Unit (LOMWRU), Mahosot Hospital, Vientiane, Laos; 2020.
41. Dance DA, Davong V, Soeng S, et al. Trimethoprim/sulfamethoxazole resistance in *Burkholderia pseudomallei*. *Int J Antimicrob Agents*. 2014;44(4):368-9.
42. Tantibhedhyangkul W, Angelakis E, Tongyoo N, et al. Intrinsic fluoroquinolone resistance in *Orientia tsutsugamushi*. *Int J Antimicrob Agents*. 2010;35(4):338-41.
43. Phuklia W, Panyanivong P, Sengdetka D, et al. Novel high-throughput screening method using quantitative PCR to determine the antimicrobial susceptibility of *Orientia tsutsugamushi* clinical isolates. *J Antimicrob Chemother*. 2019;74(1):74-81.
44. Boss J, Dance DAB, Chanthongthip A, et al. Antimicrobial susceptibility testing of *Leptospira* spp. in the Lao People's Democratic Republic using disk diffusion. *Am J Trop Med Hyg*. 2019;100(5):1073-8.
45. Iwanaga M, Insisiengmay S, Higa N, et al. Tetracycline resistant and polymyxin B sensitive *Vibrio cholerae* O1 El Tor isolated from the recent epidemics. *Jpn J Trop Med Hyg*. 2000;28(1):15-8.

46. Phantouamath B, Sithivong N, Sisavath L, et al. Transition of drug susceptibilities of *Vibrio cholerae* O1 in Lao People's Democratic Republic. *Southeast Asian J Trop Med Public Health*. 2001;32(1):95-9.
47. Iwanaga M, Toma C, Miyazato T, et al. Antibiotic resistance conferred by a class I integron and SXT constin in *Vibrio cholerae* O1 strains isolated in Laos. *Antimicrob Agents Chemother*. 2004;48(7):2364-9.
48. Rattanavong S, Dance DA, Davong V, et al. Group A streptococcal strains isolated in Lao People's Democratic Republic from 2004 to 2013. *Epidemiol Infect*. 2016;144(8):1770-3.
49. Vannarath S, Vilaichone RK, Rasachak B, et al. Antibiotic Resistant Pattern of *Helicobacter pylori* Infection Based on Molecular Tests in Laos. Asian Pacific journal of cancer prevention : APJCP. 2016;17(1):285-7.
50. Cheong E RT, Rattanavong S, et al. Clostridium difficile infection in the Lao People's Democratic Republic: first isolation and review of the literature. BMC infectious diseases. 2017;17(1):635.
51. Batty EM, Cusack TP, Thaipadungpanit J, et al. The spread of chloramphenicol-resistant Neisseria meningitidis in Southeast Asia. *Int J Infect Dis*. 2020;95:198-203.
52. Iem V, Somphavong S, Buisson Y, et al. Resistance of *Mycobacterium tuberculosis* to antibiotics in Lao PDR: first multicentric study conducted in 3 hospitals. BMC infectious diseases. 2013;13:275.
53. Iem V, Dean A, Zignol M, et al. Low prevalence of MDR-TB in Lao PDR: results from the first national anti-tuberculosis drug resistance survey. *Trop Med Int Health*. 2019;24(4):421-31.
54. Somphavong S BJ, Gauthier M, et al. First insights into the genetic characteristics and drug resistance of Mycobacterium tuberculosis population collected during the first national tuberculosis prevalence survey of Lao PDR (2010-2011). BMC infectious diseases. 2019;19(1):851.
55. Boonmar S, Markvichitr K, Chaunchom S, et al. *Salmonella* prevalence in slaughtered buffaloes and pigs and antimicrobial susceptibility of isolates in Vientiane, Lao People's Democratic Republic. *J Vet Med Sci*. 2008;70(12):1345-8.
56. Boonmar S, Morita Y, Pulsrikarn C, et al. *Salmonella* prevalence in meat at retail markets in Pakse, Champasak Province, Laos, and antimicrobial susceptibility of isolates. *J Glob Antimicrob Resist*. 2013;1(3):157-61.
57. Sinwat N, Angkittitrakul S, Coulson KF, et al. High prevalence and molecular characteristics of multidrug-resistant *Salmonella* in pigs, pork and humans in Thailand and Laos provinces. *J Med Micrbiol*. 2016;65(10):1182-93.
58. Sunn K. Phenotypic and genotypic characteristics of Extended-spectrum Beta-lactamase (ESBL) production and Colistin-resistance in *Salmonella enterica* and *Escherichia coli* isolated from pigs and their meat products in the border provinces between Thailand and Cambodia, Lao PRD and Myanmar [Science in Veterinary Science and technology]. Faculty of Veterinary Science: Chulalongkorn University, Thailand; 2018.
59. Chang K. Epidemiology of Extended Spectrum Beta-latamase in Lao PDR [Infectious disease fellowship]. Faculty of postgraduate studies University of Health Sciences, Lao PDR; 2016.
60. Olaitan AO, Thongmalayvong B, Akkhavong K, et al. Clonal transmission of a colistin-resistant *Escherichia coli* from a domesticated pig to a human in Laos. *J Antimicrob Chemother*. 2015;70(12):3402-4.

61. Thu WP SN, Bitrus AA, Angkittitrakul S, Prathan R, Chuanchuen R. Prevalence, antimicrobial resistance, virulence gene, and class 1 integrons of *Enterococcus faecium* and *Enterococcus faecalis* from pigs, pork and humans in Thai-Laos border provinces. J Glob Antimicrob Resist. 2019;18:130-8.

62. Syhakhang L, Freudenthal S, Tomson G, et al. Knowledge and perceptions of drug quality among drug sellers and consumers in Lao PDR. *Health Policy Plan*. 2004;19(6):391-401.

63. Keohavong B, Syhakhang L, Sengaloundeth S, et al. Rational use of drugs: prescribing and dispensing practices at public health facilities in Lao PDR. *Pharmacoepidemiol Drug Saf*. 2006;15(5):344-7.

64. Sihavong A, Lundborg CS, Syhakhang L, et al. Antimicrobial self medication for reproductive tract infections in two provinces in Lao People's Democratic Republic. *Sex Transm Infect*. 2006;82(2):182-6.

65. Khennavong M, Davone V, Vongsouvath M, et al. Urine antibiotic activity in patients presenting to hospitals in Laos: implications for worsening antibiotic resistance. *Am J Trop Med Hyg*. 2011;85(2):295-302.

66. Sihavong A LC, Syhakhang L, et al. Community perceptions and treatment-seeking behaviour regarding reproductive tract infections including sexually transmitted infections in Lao PDR: A qualitative study. *J Biosoc Sci*. 2011;43(3):285-303.

67. Quet F, Vlieghe E, Leyer C, et al. Antibiotic prescription behaviours in Lao People's Democratic Republic: a knowledge, attitude and practice survey. *Bull World Health Organ*. 2015;93(4):219-27.

68. Phonlavong C, Kitikannakorn N. General population's knowledge, beliefs, and care-seeking behavior about antibiotics: a cross-sectional survey in Lao People's Democratic Republic. *Thai J Pharm Prac*. 2019;10(2):411-20.

69. Keohavong B, Vonglokham M, Phoummalaysith B, et al. Antibiotic prescription for under-fives with common cold or upper respiratory tract infection in Savannakhet Province, Lao PDR. *Trop Med Health*. 2019;47(1):16.

70. Tabernero P, Swamidoss I, Mayxay M, et al. A random survey of the prevalence of falsified and substandard antibiotics in the Lao PDR. *J Antimicrob Chemother*. 2019;74(8):2417-25.

71. Haenssgen MJ CN, Zanello G, et al. Antibiotic knowledge, attitudes and practices: new insights from cross-sectional rural health behaviour surveys in low-income and middle-income South-East Asia. *BMJ Open*. 2019;9(8):e028224.

72. Haenssgen MJ CN, Xayavong T, et al. Precarity and clinical determinants of healthcare-seeking behaviour and antibiotic use in rural Laos and Thailand. *BMJ Glob Health*. 2020;5(12):e003779.

73. LOMWRU. Data from: Hospital antimicrobial point prevalence survey 2017-2018. Lao-Oxford Mahosot Hospital Wellcome Trust Research Unit (LOMWRU), Mahosot Hospital, Vientiane, Laos; 2018.

74. Labatut F, Suter I. The Veterinary Care of Domesticated Elephants in Laos by a Mobile Veterinary Unit. *GAJAH*. 2010(32):21-6.

75. Theungphachan T. “Country Report: Lao PDR.” Livestock & Fisheries Products Quality Assurance Unit, NAHC, DLF 2012 [Access date 09 April 2021]. Available from: http://webcache.googleusercontent.com/search?q=cache:http://lad.nafri.org.la/fulltext/3783-0.pdf.

76. Phanthavong S. Study on treatment of demodex canine disease of dogs. University of Agriculture: University of Agriculture, Laos; 2016.

77. Innoula N. Study on the treatment of canine parvoviral enteritis by using antibiotic University of Agriculture: University of Agriculture, Laos; 2017.
78. Boudakham M. Study on the effectives of antibiotic for canine distemper University of Agriculture: University of Agriculture, Laos; 2017.
79. Keovilay P. Effectiveness of antibiotics on enteritis diseases in dogs. University of Agriculture: University of Agriculture, Laos; 2017.
