# Supplementary figures and images for: Antimicrobial use and resistance data in human and animal sectors in the Lao PDR: evidence to inform policy
Source: BMJ Glob Health. 2021 Dec 1;6(12):e007009. doi: 10.1136/bmjgh-2021-007009 (PMC8638151; doi:10.1136/bmjgh-2021-007009)

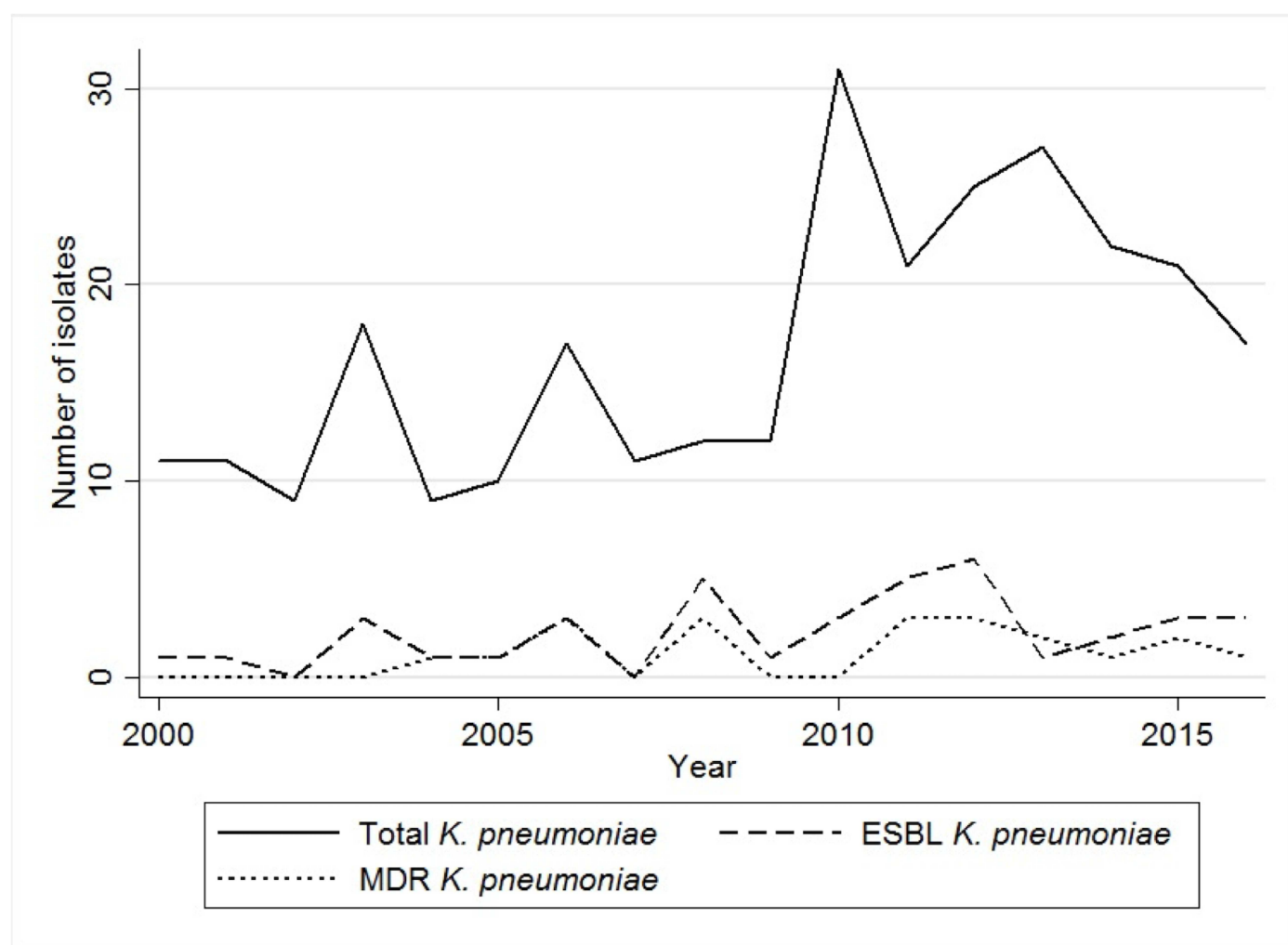

Supplement: Supplementary data [file bmjgh-2021-007009supp002.pdf]

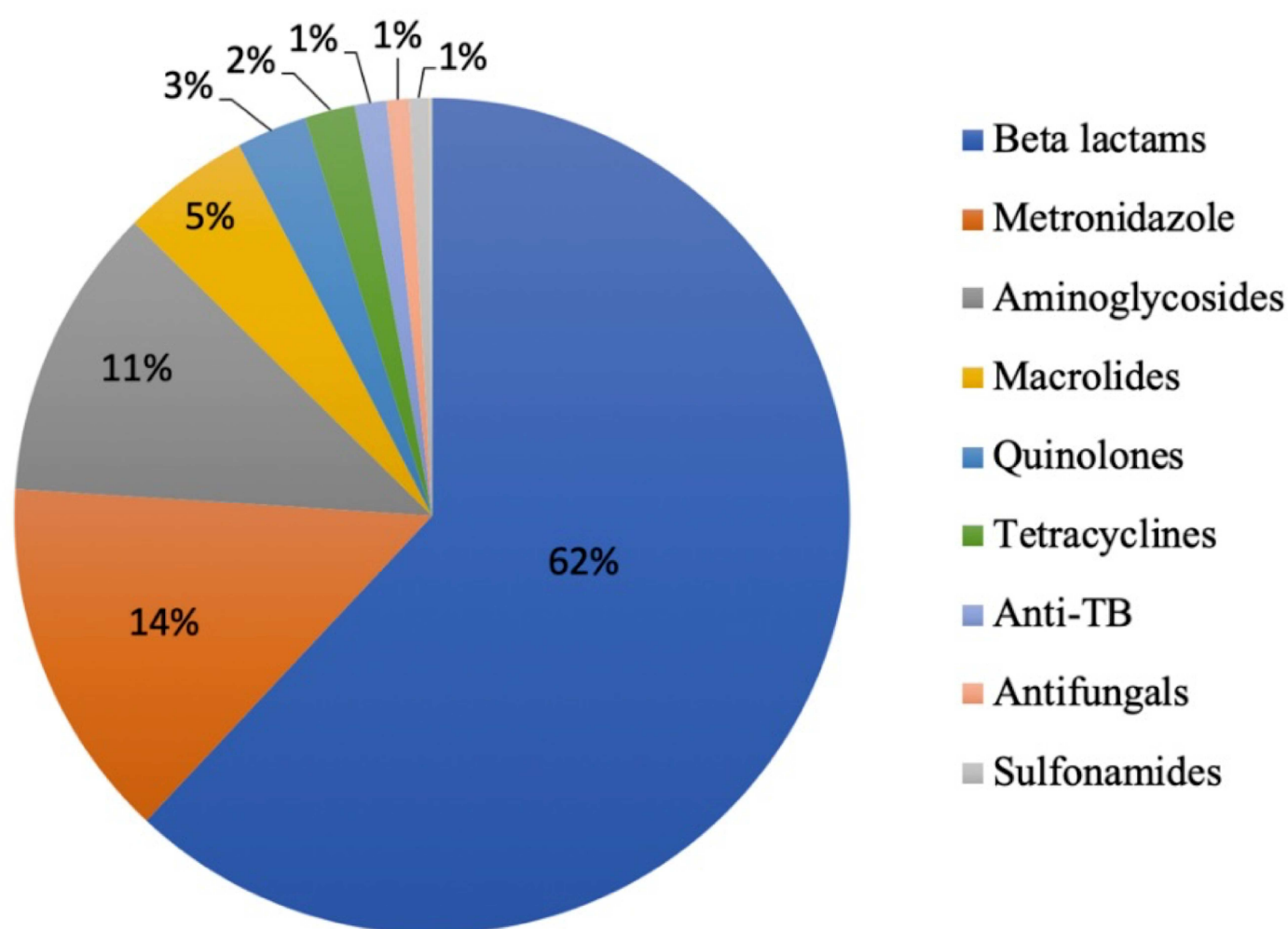

Supplement: Supplementary data [file bmjgh-2021-007009supp003.pdf]

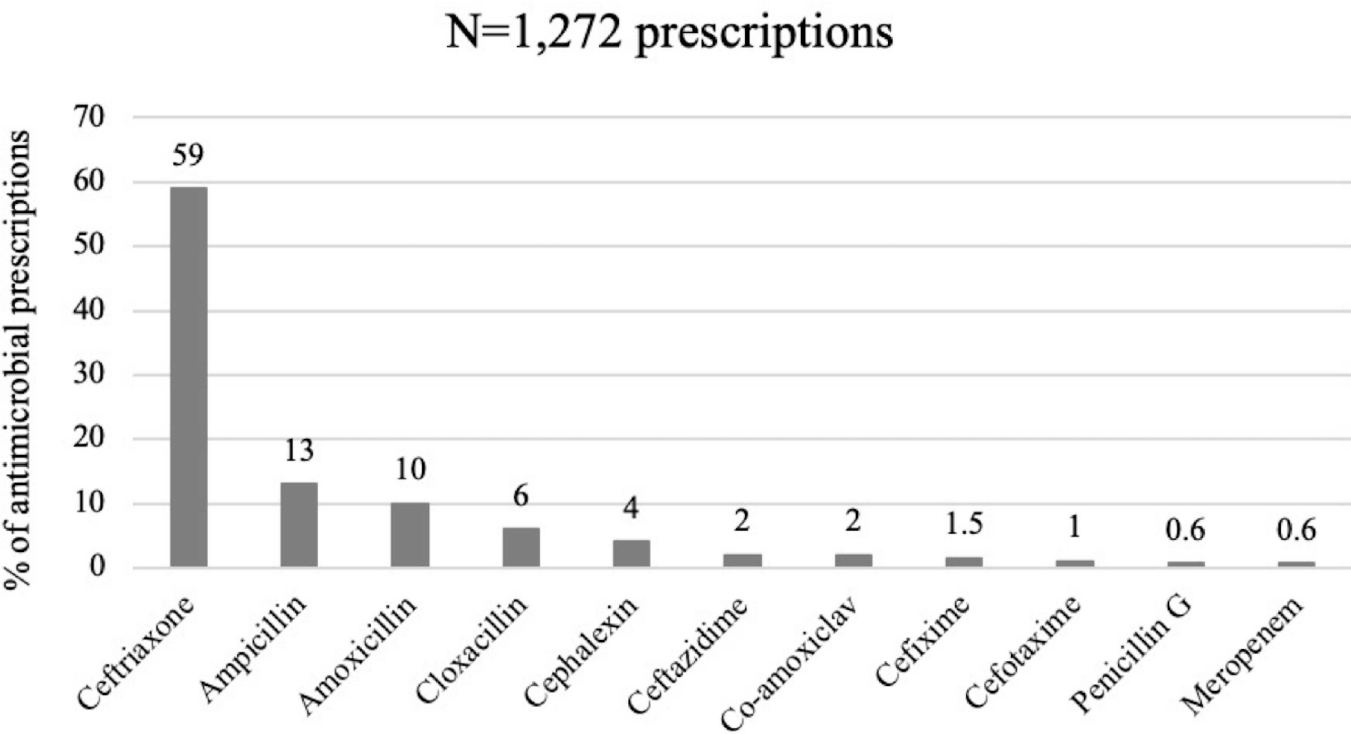

Supplement: Supplementary data [file bmjgh-2021-007009supp004.pdf]
